# Rescue Everolimus Post Lung Transplantation is Not Associated With an Increased Incidence of CLAD or CLAD-Related Mortality

**DOI:** 10.3389/ti.2023.10581

**Published:** 2023-02-07

**Authors:** Steven Ivulich, Miranda Paraskeva, Eldho Paul, Carl Kirkpatrick, Michael Dooley, Gregory Snell

**Affiliations:** ^1^ The Alfred Hospital, Melbourne, VIC, Australia; ^2^ Centre for Medication Use and Safety, Monash University, Melbourne, VIC, Australia; ^3^ Public Health and Preventative Medicine, Monash University, Melbourne, VIC, Australia

**Keywords:** lung transplantation, everolimus, bronchiolitis obliterans syndrome, restrictive allograft syndrome, calcineurin inhibitor, chronic lung allograft dysfunction

## Abstract

Everolimus (EVE) has been used as a calcineurin inhibitor (CNI) minimization/ elimination agent or to augment immunosuppression in lung transplant recipients (LTR) with CNI-induced nephrotoxicity or neurotoxicity. The long-term evidence for survival and progression to chronic lung allograft dysfunction (CLAD) is lacking. The primary aim was to compare survival outcomes of LTR starting EVE-based immunosuppression with those remaining on CNI-based regimens. The secondary outcomes being time to CLAD, incidence of CLAD and the emergence of obstructive (BOS) or restrictive (RAS) phenotypes. Single center retrospective study of 91 LTR starting EVE-based immunosuppression matched 1:1 with LTR remaining on CNI-based immunosuppression. On multivariate analysis, compared to those remaining on CNI-based immunosuppression, starting EVE was not associated with poorer survival [HR 1.04, 95% CI: 0.67–1.61, *p* = 0.853], or a statistically significant faster time to CLAD [HR 1.34, 95% CI: 0.87–2.04, *p* = 0.182]. There was no difference in the emergence of CLAD (EVE, [*n* = 57, 62.6%] vs. CNI-based [*n* = 52, 57.1%], *p* = 0.41), or the incidence of BOS (*p* = 0.60) or RAS (*p* = 0.16) between the two groups. Introduction of EVE-based immunosuppression does not increase the risk of death or accelerate the progression to CLAD compared to CNI-based immunosuppression.

## Graphical Abstract

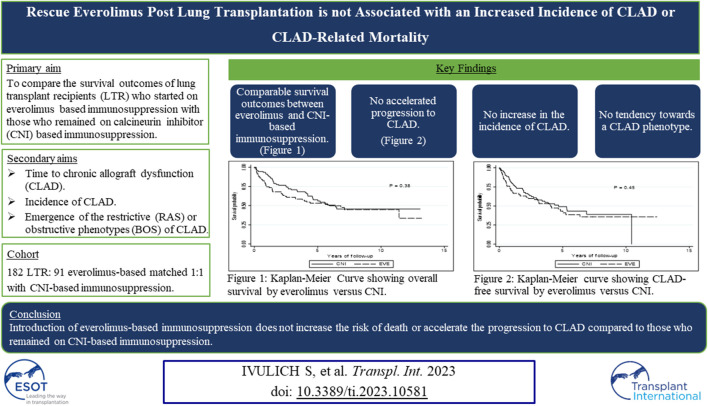

## Introduction

Chronic lung allograft dysfunction (CLAD) remains the limiting factor for long-term survival after lung transplantation (LTx), with poorer outcomes compared to other solid organ transplants (SOTs) and a median survival of 6.5 years (1). Despite the evolution of perioperative and post-operative management strategies over the last 2 decades, immunosuppressive regimens have remained relatively unchanged. Traditional regimens typically consist of a calcineurin inhibitor (CNI), such as tacrolimus or ciclosporin, an antiproliferative (mycophenolate or azathioprine) and a corticosteroid. Everolimus (EVE), a mammalian target of rapamycin (mTOR) inhibitor has only recently been considered a potential maintenance immunosuppressant, particularly for those with CNI induced nephrotoxicity or neurotoxicity (2–5).

EVE has unique pharmacological actions distinct from other currently available immunosuppressant classes and provides a novel potential therapeutic role in LTx (6). The use of mTOR inhibitors may reduce *Cytomegaloviru*s (CMV) infection (7) and could have some anti-cancer effect due to its anti-angiogenic properties (8, 9). Further, the antifibrotic effect of EVE has been postulated to be beneficial in those with CLAD (10).

However, EVE also has potentially problematic effects in the LTx setting. In particular, its use is not recommended in the early transplant period due to the risk of wound and anastomotic dehiscence (10). In addition, EVE has infrequently been associated with pulmonary toxicity, in particular an interstitial pneumonitis, which may be difficult to distinguish from CLAD (11).

The benefits of EVE for renal preservation after LTx are well documented in several randomized trials (4, 12). Although EVE-based immunosuppression has been shown to be effective in preserving short-term renal function, the long-term benefits have not been maintained (13).

The potential of EVE to prevent CLAD is less well studied. A study by Streuber et al. investigated the impact of EVE on rejection outcomes after LTx. Freedom from bronchiolitis obliterans syndrome (BOS) was investigated in a prospective, randomized trial comparing mycophenolate to everolimus. However, the investigators were unable to prove a difference due to the high withdrawal rate.

A current trial is investigating the immunomodulatory effects of tacrolimus, everolimus and alemtuzumab on kidney function, allograft acceptance and the risk of CLAD. This study is investigating the impact of low-dose everolimus with tacrolimus and alemtuzumab in preventing CNI-driven kidney damage with the potential advantage of further reducing tacrolimus target levels and reducing CLAD (14).

Despite these recent studies, the long-term impact of its use as maintenance immunosuppression on survival and CLAD has yet to be determined. In this retrospective case-controlled study, we compare LTR who started on an EVE-based maintenance immunosuppression regimen with those who remained on a CNI-based regimen. The primary aim of the study was to assess the effect of these immunosuppression approaches on survival, with the secondary outcome being time to CLAD. In addition, we investigated whether EVE-based maintenance immunosuppression contributes to the development of the different CLAD phenotypes: BOS or restrictive allograft syndrome (RAS).

## Materials and Methods

### Patient Population

Between 2005 and 2018, 1100 LTx were undertaken, with institutional recipient management and donor selection protocols described previously (15, 16). All recipients received standard triple immunosuppression with tacrolimus or ciclosporin (pre-2008), azathioprine or mycophenolate mofetil and corticosteroids. All individuals prescribed EVE were considered for inclusion. Excluded were those: lost to follow up, early discontinuation (duration of therapy <3 months) or previous sirolimus therapy.

Recipients on EVE were matched 1:1 with those who remained on CNI-based immunosuppression based on: transplant date, procedure, age at transplant, sex, and underlying diagnosis. After matching, survival and time to CLAD outcomes were calculated from the date of EVE commencement. If a LTR who remained on CNI-based immunosuppression did not survive to the date of starting EVE, they were excluded from further analysis. The final cohort consisted of 182 LTR (91 EVE recipients with 91 CNI controls).

### Definition of Rejection

Acute cellular rejection was defined as changes on transbronchial biopsy of ≥ International Society for Heart and Lung Transplantation (ISHLT) Grade 2, or in the absence of a biopsy an otherwise unexplained drop in lung function treated with intravenous corticosteroid (17, 18). Acute antibody-mediated rejection was diagnosed and managed according to Alfred Hospital protocols (19).

### Spirometric Monitoring, Definition, and Treatment of CLAD

All LTR living within 300 km of our centre underwent indefinite long-term follow-up with regular spirometry. Spirometry at time of starting on EVE, 1-year following change and at time of diagnosis of CLAD was investigated for its impact on survival and time to CLAD.

CLAD was defined as an irreversible decline in forced expiratory volume in 1 s (FEV_1_) to <80% of baseline (the mean of the two best post-LTx measurements, obtained at least 3 weeks apart with or without a decline in forced vital capacity [FVC]) (20). The phenotypes of CLAD were defined as either BOS (FEV_1_/FVC <0.7 and FVC ≥80% predicted baseline FVC at CLAD onset) or RAS (FEV_1_/FVC ≥0.7 and FVC <80% predicted baseline FVC at CLAD onset) (21, 22). Whilst total lung capacity (TLC) is not routinely undertaken and was not available on all recipients to allow its use in the definition, we utilised the spirometric definition detailed above to define RAS as detailed in the published consensus guidelines (21, 22). Declines in lung function/CLAD were treated according to the standard protocols of the time (18). For this analysis CLAD status, staging and phenotype were redefined as per ISHLT criteria (20, 23).

### General Management Strategy for Renal Impairment

Induction therapy with the IL-2 receptor blocker, basiliximab, was given as a CNI sparing agent to LTR who were identified pre-transplant as being at higher risk of developing post-LTx renal dysfunction (*n* = 73). Subsequent strategies for LTR with renal impairment involved CNI reduction (*n* = 47) or elimination (*n* = 44); control of hypertension, diabetes, and cholesterol; and initiation of EVE (22). For LTR receiving EVE in combination with a CNI for a renal indication, further increases in serum creatinine would warrant eventual withdrawal of the CNI.

### General Management Strategy for CMV

CMV prophylaxis, monitoring and treatment strategies are described elsewhere (24). Immediate post-transplant prophylaxis for all patients at risk of CMV infection received at least 7 days of intravenous ganciclovir followed by valganciclovir for a duration determined by risk category. Severe CMV infection or CMV reactivation was defined as >10,000 IU/mL in the blood or CMV >50,000 IU/mL in the bronchoalveolar lavage (BAL).

### EVE Indications, Dosing, TDM and Utilization Strategy

EVE was utilized in the setting of failure of first-line immunosuppressive strategies, e.g., significant renal impairment, CNI-neurotoxicity, malignancy (25, 18). EVE was prescribed with or without a CNI, determined by the degree of CNI intolerance. As per unit protocol, EVE was typically commenced at a dose of 0.25–0.5 mg twice daily with halving of the CNI dose. If EVE was to be used in conjunction with a CNI (minimization strategy) a trough concentration of 3–5 ng/mL for EVE and 4–6 ng/mL for tacrolimus would be targeted. If CNI cessation was planned (elimination strategy), a trough concentration of 5–7 ng/mL for EVE was targeted. Whenever EVE was utilized as part of a CNI elimination strategy, the CNI was ceased when EVE trough concentration was ≥3 ng/mL.

### Statistical Analyses

Continuous variables were summarized using means and standard deviations (SD) or medians and interquartile ranges (IQR) wherever appropriate. Categorical variables were expressed as counts and percentages. Overall survival was defined as the time from the date of starting EVE to the date of death or last follow-up. Time to CLAD was calculated from the date of starting EVE to the date of diagnosis of CLAD.

Univariate and multivariate analyses for overall survival and time to CLAD were performed using Cox proportional hazards regression with results reported as hazard ratios (HR) and 95% confidence intervals (95% CI). Variables with a *p* < 0.05 on univariable analyses or those deemed clinically relevant were considered for inclusion in the multivariable models.

To account for any possible imbalance between groups due to differences in baseline demographics and the evolution of treatments, propensity scores were included as an additional covariate in the regression models. Propensity score matching was also used to reduce selection bias from confounding factors between the EVE or CNI-based immunosuppression group. The individual propensities for being in the EVE group were estimated with the use of a multivariable logistic regression model that included date of transplant, age at transplant, sex, azithromycin and CMV reactivation as the predictor variables. All calculated p-values were two-tailed and a *p* < 0.05 indicated statistical significance. Statistical analyses were performed using SAS version 9.4 (SAS Institute, Cary, NC, United States).

Changes in estimated glomerular filtrate rate (eGFR) over time (time of LTx, EVE commencement and 1-year post) was assessed using linear mixed models fitting main effects for time, group (EVE or CNI) and their two-way interactions.

### Ethics Approval

The study was approved by the Alfred Hospital (252-12, 30 May 2012) and Monash University Ethics Committees (252-12, 19 April 2017).

## Results

### Patient Characteristics and Indications for EVE Use

Baseline demographics are described in Table 1. The most common indication for starting EVE was renal impairment (79%), followed by malignancy (8%), neurotoxicity (7%), intolerance to CNI (4%) and recurrent CMV (2%). The median time from LTx to initiation of EVE was 334 days [IQR: 155-604], with the median time of follow up for all LTR included being 1881 days [IQR: 993–2970].

**TABLE 1:** Demographics.

	EVE (*n* = 91)	CNI (*n* = 91)	*p*-value
Characteristic			
Age (yr), mean	51.64 ± 13.87	50.81 ± 13.87	0.16
Gender: male, n (%)	49 (53.8)	49 (53.8)	1.00
Body mass index (mean ± SD)	25.00 ± 4.45	25.93 ± 5.28	0.15
Indication for transplantation, n (%)			
Chronic obstructive pulmonary disease	43 (47.2)	43 (47.2)	1.00
Cystic fibrosis	19 (20.9)	19 (20.9)	1.00
Interstitial lung disease	19 (20.9)	19 (20.9)	1.00
Pulmonary hypertension	6 (6.6)	6 (6.6)	1.00
Other	4 (4.4)	4 (4.4)	1.00
Transplantation type, n (%)			
Bilateral sequential lung	81 (89.0)	81 (89.0)	1.00
Single lung	9 (9.9)	9 (9.9)	1.00
Heart and lung	1 (1.1)	1 (1.1)	1.00
Maintenance Immunosuppression, n (%)^a^			
Tacrolimus	75 (82.4)	82 (90.1)	0.07
Ciclosporin	16 (17.6)	9 (9.9)	0.07
Mycophenolate	38 (41.7)	33 (36.3)	0.44
Azathioprine	34 (37.4)	52 (57.1)	0.009
No antimetabolite	19 (20.9)	6 (6.6)	0.014
Rejection			
Acute Rejection ^b^	13 (14.3)	15 (16.5)	0.66
Diagnosis of CLAD^c^	57 (62.6)	52 (57.1)	0.41
RAS	27 (29.7)	19 (20.9)	0.16
BOS	30 (33.0)	33 (36.3)	0.60

Abbreviations: ACR, acute cellular rejection; BOS, bronchiolitis obliterans syndrome; CLAD, chronic lung allograft dysfunction; EVE, everolimus; ISHLT, international society for heart and lung transplantation; RAS, restrictive allograft syndrome.

^a^ Maintenance immunosuppression at time of starting on EVE.

^b^ Episode of ISHLT graded ≥2 ACR pre or post starting on EVE.

^c^ Diagnosis of CLAD pre or post starting on EVE.

### Overall Survival

**FIGURE 1:**
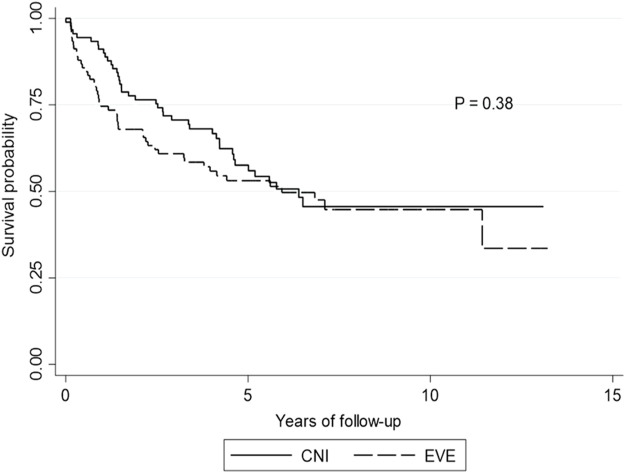
Kaplan-Meier curve for overall survival. Kaplan-Meier curve showing overall survival by calcineurin inhibitor versus everolimus. *p*-value is calculated from a log rank test comparing the entire survival experience between the two groups (calcineurin inhibitor versus everolimus). Abbreviations: CNI, calcineurin inhibitor; EVE, everolimus.

The median survival for the entire cohort was 1881 days [IQR: 993–2970], (EVE: 1869 days [IQR: 910–3185] vs. CNI: 1944 days [IQR: 1196–2850]). One, three- and five-year survival was 80.2%, 68.1% and 59.9%, respectively. There was no difference in overall mortality between the groups (48% EVE vs. 45% CNI, *p* = 0.648) (Figure 1).

#### Univariate Analysis

On univariate analysis (Table 2), compared to CNI-based immunosuppression commencement of EVE based immunosuppression was not associated with a statistically significant poorer survival outcome [HR 1.15, 95% CI: 0.75–1.76, *p* = 0.514]. After adjusting for propensity score, survival outcomes were also comparable to those who remained on CNI-based immunosuppression [HR 1.12, 95% CI: 0.74–1.70, *p* = 0.577].

**TABLE 2:** Univariate analysis: Summary of effects of different coviarates on survival.

Summary of effects of different covariates on survival (*n* = 182)			
		Hazard ratio	*p*-value
Demographics	Male	1.10 (0.72–1.67)	0.668
	Age	1.00 (0.99–1.02)	0.742
	Body mass index	0.98 (0.94–1.02)	0.344
	Surgical Procedure		
	BSLTx	0.98 (0.56–1.73)	0.954
	SLTx	0.84 (0.47–1.52)	0.566
Diagnosis	Cystic Fibrosis	0.81 (0.50–1.34)	0.422
	Chronic obstructive pulmonary disease	1.25 (0.83–1.90)	0.290
	Interstitial lung disease	0.87 (0.51–1.48)	0.610
	Pulmonary Hypertension	0.51 (0.22–1.18)	0.118
	Retransplant	1.49 (0.43–5.12)	0.53
	Other	2.08 (0.84–5.16)	0.113
Everolimus	Use of EVE	1.15 (0.75–1.76)	0.514
	Time of Transplant to Initiation	0.99 (0.98–0.99)	<0.0001
	Discontinuation of EVE	1.99 (1.11–3.54)	0.020
	Indication for EVE	0.91 (0.56–1.47)	0.699
	EVE Level	1.06 (0.96–1.16)	0.282
Immunosuppression^a^	Tacrolimus	1.32 (0.79–2.19)	0.290
	Ciclosporin	0.83 (0.48–1.42)	0.492
	Azathioprine	1.14 (0.75–1.72)	0.542
	Mycophenolate	0.62 (0.39–0.99)	0.047
Indication	Renal preservation	1.65 (0.87–3.12)	0.128
CLAD	Existence of CLAD^b^	3.73 (2.06–6.75)	<0.0001
	CLAD phenotype	0.59 (0.36–0.96)	0.033
	CLAD at time of starting EVE	0.84 (0.39–1.82)	0.657
	FER at diagnosis of CLAD	0.98 (0.96–0.98)	0.024
	Azithromycin prophylaxis	0.52 (0.30–0.91)	0.019
Spirometry	FEV_1_: Time of starting EVE (measured)	0.70 (0.56–0.87)	0.002
	FEV_1_: Time of starting EVE (percentage)	0.98 (0.97–0.99)	0.0001
	FVC: Time of starting EVE (measured)	0.79 (0.66–0.96)	0.018
	FVC: Time of starting EVE (percentage)	0.98 (0.98–0.99)	0.005
	FEV_1_:1-year post starting EVE (measured)	0.49 (0.36–0.66)	<0.0001
	FEV_1_:1-year post starting EVE (percentage)	0.97 (0.96–0.98)	<0.0001
	FVC: 1-year post starting EVE (measured)	0.69 (0.53–0.90)	0.006
	FVC: 1-year post starting EVE (percentage)	0.97 (0.96–0.98)	<0.0001
*Cytomegalovirus*	*Cytomegalovirus* reactivation^c^	1.33 (0.84–2.08)	0.219

Abbreviations: BSLTx, bilateral sequential lung transplant; CLAD, chronic lung allograft dysfunction; EVE, everolimus; FER, forced expiratory ratio; FEV_1_, forced expiratory volume in 1 s; FVC, forced vital capacity; SLTx, single lung transplant.

^a^ Maintenance immunosuppression at time of starting EVE.

^b^ Diagnosis of BOS or RAS pre or post starting EVE.

^c^ *Cytomegalovirus* reactivation post starting EVE.

On univariate analysis, starting on EVE for a renal indication had no impact on survival [HR 1.65, 95% CI: 0.87–3.12, *p* = 0.128]. However, LTR who started EVE for renal preservation were more likely to have a faster progression to CLAD [HR 1.98, 95% CI: 1.09–3.59, *p* = 0.024].

The development of CLAD at any time point was associated with mortality [HR 3.73, 95% CI: 2.06–6.75, *p* = 0.0001]. However, CLAD diagnosed prior to starting EVE was not a predictor of death (*p* = 0.657). Recipients who developed RAS had a lower risk of death than those with BOS (HR 0.591 [0.365–0.959], *p* = 0.033).

All spirometric indices measured at time of EVE initiation and 1 year following change were significant predictors of survival (Table 2). The forced expiratory ratio (FEV_1_/FVC) at time of CLAD diagnosis was a predictor of improved survival (HR 0.978 [0.959–0.997], *p* = 0.024).

#### Timing of EVE Commencement

The timing of starting EVE did not impact survival. LTR who started EVE prior to 1-year post LTx had similar survival outcomes to those who started after 1-year post LTx [HR 0.97, 95% CI: 0.63–1.49, *p* = 0.88].

### Time to CLAD

#### Univariate Analysis

**FIGURE 2:**
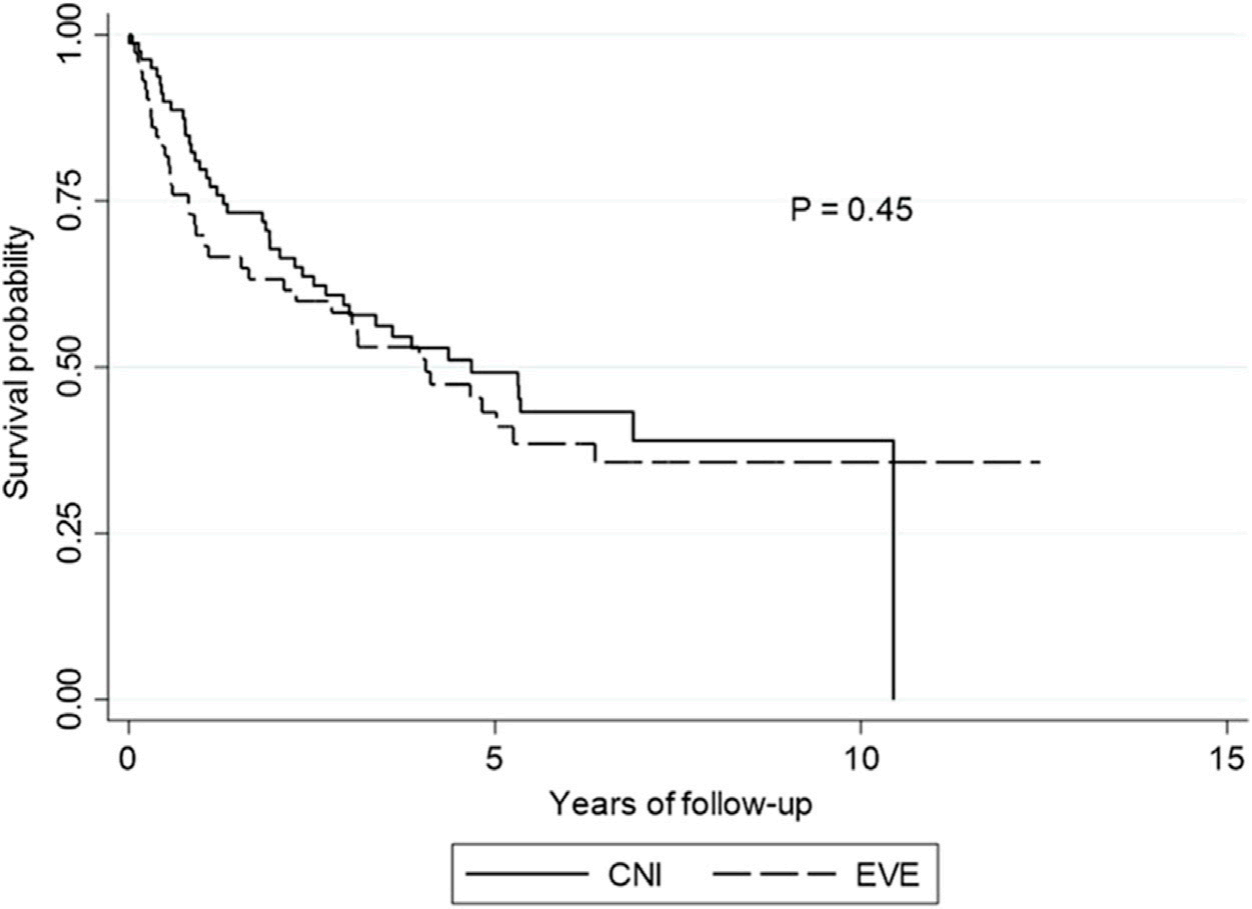
Kaplan-Meier curve for CLAD-free survival. Kaplan-Meier curve showing CLAD-free survival by calcineurin inhibitor versus everolimus. *p*-value is calculated from a log rank test comparing the CLAD-free survival between the two groups (calcineurin inhibitor versus everolimus). Abbreviations: CM, calcineurin inhibitor; EVE, everolimus.

Following the univariate analysis, compared to those remaining on CNI-based immunosuppression initiation of EVE-based immunosuppression did not statistically accelerate the progression to CLAD diagnosis [HR 1.27, 95% CI: 0.87–1.86, *p* = 0.208] (Figure 2). After adjusting for propensity score, compared to CNI-based immunosuppression, EVE was not associated with a faster time to CLAD [HR 1.26, 95% CI: 0.89–1.77, *p* = 0.190]. All spirometric indices measured at time of starting EVE and 1 year following change were predictors of time to CLAD (Table 3).

**TABLE 3:** Univariate analysis: Summary of effects of different covariates on time to CLAD.

Summary of effects of different covariates on time to CLAD (*n* = 182)			
		Hazard ratio	*p*-value
Demographics	Male	1.22 (0.81–1.82)	0.342
	Age	1.01 (0.99–1.02)	0.338
	Body mass index	1.02 (0.98–1.06)	0.315
	Surgical Procedure		
	BSLTx	0.88 (0.52–1.51)	0.653
	SLTx	1.05 (0.56–1.96)	0.880
Diagnosis	Cystic Fibrosis	0.52 (0.30–0.90)	0.019
	Chronic obstructive pulmonary disease	1.46 (0.98–2.17)	0.060
	Interstitial lung disease	1.03 (0.64–1.65)	0.902
	Pulmonary hypertension	1.08 (0.52–2.22)	0.838
	Re-Transplant	1.27 (0.22–7.45)	0.789
	Other	1.01 (0.25–4.13)	0.991
EVE	Use as an Immunosuppressant	1.27 (0.87–1.86)	0.208
	Time of Transplant to Initiation	1.00 (1.00–1.00)	0.446
	Discontinuation of EVE	1.46 (0.84–2.53)	0.178
	EVE Level	1.03 (0.91–1.16)	0.677
Immunosuppression^a^	Tacrolimus	0.86 (0.51–1.46)	0.580
	Ciclosporin	1.08 (0.61–1.92)	0.782
	Azathioprine	0.99 (0.68–1.45)	0.974
	Mycophenolate	0.76 (0.51–1.13)	0.175
	Tacrolimus Level	1.04 (0.97–1.11)	0.319
	Ciclosporin Level	1.00 (1.00–1.00)	0.725
Indication	Renal preservation	1.98 (1.09–3.59)	0.024
CLAD	Azithromycin prophylaxis	1.22 (0.66–2.27)	0.522
Spirometry	FEV_1_: Time of starting EVE (measured)	0.67 (0.53–0.86)	0.001
	FEV_1_: Time of starting EVE (percentage)	0.98 (0.97–0.99)	0.003
	FVC: Time of starting EVE (measured)	0.84 (0.69–1.01)	0.064
	FVC: Time of starting EVE (measured)	0.99 (0.98–1.00)	0.012
	FEV_1_: 1-year post starting EVE (measured)	0.57 (0.44–0.73)	<0.0001
	FEV_1_: 1-year post starting EVE (percentage)	0.97 (0.97–0.98)	<0.0001
	FVC: 1-year post starting EVE (measured)	0.67 (0.54–0.83)	0.0003
	FVC: 1-year post EVE starting (percentage)	0.97 (0.96–0.98)	<0.0001
*Cytomegalovirus*	*Cytomegalovirus* Reactivation ^b^	1.28 (0.86–1.92)	0.220

Abbreviations: BSLTx, bilateral sequential lung transplant; CLAD, chronic lung allograft dysfunction; EVE, everolimus; FER, forced expiratory ratio; FEV_1_, forced expiratory volume in 1 s; FVC, forced vital capacity; SLTx, single lung transplant.

^a^ Maintenance immunosuppression at time of starting EVE.

^b^ *Cytomegalovirus* reactivation post starting EVE.

#### Relationship of CLAD Onset and to Time to Death

The time from diagnosis of CLAD until death was longer in the EVE group compared to the CNI-based group (1127 days, [IQR: 504–2210] vs. 427 days, [IQR: 236–1229], *p* = 0.01).

## Incidence of CLAD and CLAD Phenotypes

**FIGURE 3:**
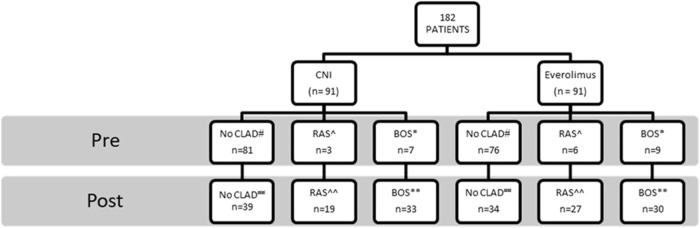
CLAD outcomes for lung transplant cohort: Everolimus versus calcineurin inhibitor group. CLAD outcomes pre and post intervention. *Comparisons between CNI and EVE groups: Pre-switch BOS* (*p* = 0.74), post-switch BOS** (*p* = 0.60), pre-switch RAS^^^ (*p* = 0.33), post-switch RAS^^^^ (*p* = 0.16), pre-switch CLAD^#^ (*p* = 021), post-switch CLAD^##^ (*p* = 0.41). Abbreviations: BOS, bronchiolitis obliterans syndrome; CLAD, chronic lung allograft syndrome; CNI, calcineurin inhibitor; RAS, restrictive allograft syndrome.

The overall incidence of CLAD at any time point was 59.8% (109/182). In the LTR who developed CLAD, 57.8% (*n* = 63) developed the BOS phenotype, whereas the remainder (42.2%, *n* = 46) developed RAS (Figure 3).

There was no difference in the emergence of CLAD between the two groups (EVE, [*n* = 57, 62.6%] vs. CNI-based [*n* = 52, 57.1%], *p* = 0.41). There was no difference in the incidence of BOS (*p* = 0.60) or RAS (*p* = 0.16) between the EVE and the CNI groups (Figure 3).

### Multivariate Analysis

#### Survival

On multivariate analysis (Table 4), starting on EVE was not associated with poorer survival [HR 1.10, 95% CI: 0.73–1.66, *p* = 0.642]. At the time of EVE introduction, FVC percentage calculated was predictive of better survival (HR 0.99 [0.98–1.00], *p* = 0.023) and both BOS (HR 4.30 [2.16–8.58], *p*=< 0.0001) and RAS (HR 2.36 [1.23–4.53], *p* = 0.010) phenotypes of CLAD were independently associated with poorer survival (Table 4). On multivariate analysis, immunosuppressant regimens containing mycophenolate were associated with improved survival (HR 0.62 [0.40–0.98], *p* = 0.043).

**TABLE 4:** Multivariate analysis—risk factors for mortality: Propensity matched pairs post conversion to everolimus.

Variable	Hazard ratio	*p*-value
EVE	1.10 (0.73–1.66)	0.642
Estimated GFR at time of starting EVE	1.00 (0.99–1.01)	0.938
FVC % predicted at time of starting EVE	0.99 (0.98–1.00)	0.023
No CLAD	REF	
BOS^a^	4.30 (2.16–8.58)	<0.0001
RAS^a^	2.36 (1.23–4.53)	0.010
Mycophenolate	0.62 (0.40–0.98)	0.043

Abbreviations: BOS, bronchiolitis obliterans syndrome; CLAD, chronic lung allograft dysfunction; EVE, everolimus; FVC, forced vital capacity; GFR, glomerular filtrate rate; RAS, restrictive allograft syndrome.

^a^ Diagnosis of BOS or RAS pre or post starting EVE.

#### Time to CLAD

On multivariate analysis, starting on EVE was not associated with a faster time to CLAD diagnosis [HR 1.34, 95% CI: 0.88–2.06, *p* = 0.176]. The variables associated with a faster time to CLAD included FVC percentage calculated at time of EVE introduction [HR 0.99, 95% CI: 0.98–1.00, *p* = 0.011], diagnosis of cystic fibrosis [HR 0.49, 95% CI: 0.25–0.97, *p* = 0.039] and a history of ISHLT grade ≥2 ACR [HR 2.37, 95% CI: 1.56–3.60, *p*=<0.0001] (Table 5).

**TABLE 5:** Multivariate analysis—risk factors for time to CLAD: Propensity matched pairs post conversion to everolimus.

Variable	Hazard ratio	*p*-value
EVE	1.34 (0.88–2.06)	0.176
Estimated GFR at time of starting EVE	1.01 (1.00–1.01)	0.143
% FVC predicted at time of starting EVE	0.99 (0.98–1.00)	0.007
Cystic Fibrosis	0.49 (0.25–0.97)	0.039
ISHLT graded ≥2 ACR^a^	2.30 (1.53–3.45)	0.0001
Mycophenolate	0.67 (0.44–1.01)	0.058

Abbreviations: ACR, acute cellular rejection; CLAD, chronic lung allograft dysfunction; EVE, everolimus; FVC, forced vital capacity; GFR, glomerular filtrate rate; ISHLT, international society for heart and lung transplantation.

^a^ Episode of ISHLT graded ≥2 ACR pre or post starting EVE.

### Preservation of Renal Function

**FIGURE 4:**
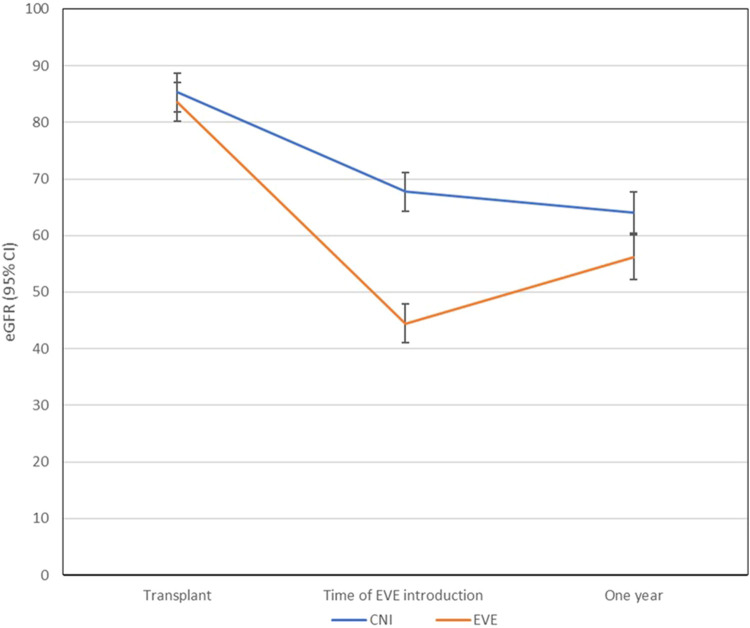
Changes in renal function (eGFR) from time to transplant to starting EVE to 1-year post. The changes in eGFR assessed over time between the two groups and their two-way interactions demonstrated significant interaction between group and time (*p* < 0.0001), suggesting that the EVE and CNI groups behaved differently over time. Abbreviations: CNI, Calcineurin inhibitor; eGFR, estimated glomet-ular filtrate rate; EVE, everolimus.

The baseline renal function between the two groups was comparable at the time of LTx (*p* = 0.478). Estimated GFR declined significantly from the time of LTx to the introduction of EVE for all included LTR (from 84.4 to 56.1 mL/min/1.73 m^2^, *p* ≤ 0.0001). At the time of commencement of EVE, there was a significant difference in estimated glomerular filtrate rate (eGFR) between the two groups (EVE, 44.5 mL/min/1.73 m^2^ vs. CNI, 67.7 mL/min/1.73 m^2^, *p* ≤ 0.0001). At 1-year follow up, the eGFR in the EVE group was significantly lower than the CNI group (EVE, 56.2 vs. CNI, 64.1 mL/min/1.73 m^2^, *p* = 0.03) (Figure 4). The changes in eGFR assessed over time between the two groups and their two-way interactions demonstrated significant interaction between group and time (*p* < 0.0001), suggesting that the EVE and CNI groups behaved differently over time (Figure 4).

### Calcineurin Inhibitor Minimization Versus Elimination

Demographics of the two EVE strategies (CNI minimization and CNI elimination) are described in Table 6. The CNI elimination strategy was not associated with poorer survival (*p* = 0.158) or a faster time to CLAD (*p* = 0.944). When comparing the eGFR changes over time, there is no statistical difference between the two groups (*p* = 0.498).

**TABLE 6:** Everolimus strategies.

Demographics	CNI minimization (*n* = 55)	CNI elimination (*n* = 36)	*p*-value
Characteristic			
Age (yr), mean	51.47 ± 14.10	51.89 ± 13.75	0.89
Gender: male, n (%)	25 (45.4)	24 (66.7)	0.047
Indication for transplantation, n (%)			
Chronic obstructive pulmonary disease	24 (43.6)	19 (52.8)	0.305
Cystic fibrosis	12 (21.8)	7 (19.4)	0.785
Interstitial lung disease	12 (21.8)	7 (19.4)	0.785
Pulmonary hypertension	4 (7.3)	2 (5.6)	0.747
Other	3 (5.5)	1 (2.8)	0.416
Transplantation type, n (%)			
Bilateral sequential lung	49 (89.0)	32 (88.9)	0.83
Single lung	5 (9.2)	4 (11.1)	0.75
Heart and lung	1 (1.8)	0 (0.0)	1.00
Maintenance Immunosuppression, n (%)^#^			
Tacrolimus	47 (85.5)	26 (72.2)	0.12
Ciclosporin	8 (14.5)	6 (16.7)	0.78
Mycophenolate	19 (34.5)	19 (52.8)	0.09
Azathioprine	25 (45.5)	9 (25.0)	0.049
Rejection			
ISHLT graded ≥2 ACR^	8 (14.5)	5 (13.9)	0.93
Diagnosis of CLAD*	34 (61.8)	23 (63.9)	0.72
RAS	18 (32.7)	13 (23.6)	0.48
BOS	16 (29.1)	10 (18.2)	0.63

Abbreviations: ACR, acute cellular rejection; BOS, bronchiolitis obliterans syndrome; CLAD, chronic lung allograft dysfunction; CNI, calcineurin inhibitor; EVE, everolimus; RAS, restrictive allograft syndrome.

^#^Maintenance immunosuppression at time of switch to EVE.

^Episode of ISHLT graded ≥ 2 ACR pre or post switch to EVE.

*Diagnosis of BOS or rCLAD pre or post switch to EVE.

### Cause of Death

There was no difference between the groups with regards to cause of death (Table 7). In particular, no difference in death due to CLAD (31.9% EVE vs. 29.6% CNI, *p* = 0.74). Additionally, whilst EVE was utilized primarily as a CNI-sparing agent to preserve renal function, there was no difference in mortality from renal failure (2.2% EVE vs. 1.1% CNI, *p* = 1.00).

**TABLE 7:** Cause of death.

Cause of death			
Cause	EVE (n = 44)	Calcineurin inhibitor (*n* = 41)	*p*-value
CLAD	29	27	0.74
Non-specific graft failure	5	4	0.66
Infection	4	5	0.57
Cerebrovascular accident	1	3	0.99
Malignancy	3	0	1.00
Renal Failure	2	1	1.00
Other: Non-adherence/Lost to follow-up	0	1	1.00

Abbreviations: CLAD, chronic lung allograft dysfunction; EVE, everolimus.

## Discussion

We believe this is the largest study examining the use of EVE-based maintenance immunosuppression in LTR. Compared to other SOTs, experience with EVE for maintenance immunosuppression in the LTx setting remains limited (27, 26). EVE was predominantly initiated in our cohort as a CNI-sparing agent in the setting of renal impairment. The most important findings from the study demonstrated that EVE can likely be safely utilized for second line immunosuppression with the aim of minimizing or eliminating CNIs without an increase in mortality, incidence of CLAD or time to CLAD.

### Overall Survival

The primary aim of our study was to determine whether LTR who started on EVE had poorer survival outcomes compared to those that remained on CNI-based immunosuppression. Overall survival, including CLAD related mortality was similar to those who remained on CNI-based immunosuppression, suggesting a similar trajectory for both groups. Our study demonstrated that EVE is a safe and effective option when prescribed for CNI intolerance, primarily nephrotoxicity or neurotoxicity as part of maintenance immunosuppressive regimens.

There has been some reluctance in using EVE as part of immunosuppressive regimens in the perioperative period due to challenges with the risk of wound dehiscence as well as destabilizing immunosuppression. While we would recommend EVE for stable long-term LTR as part of a maintenance immunosuppression regimen in LTR with CNI-induced nephrotoxicity, we would not recommend starting on EVE in the perioperative period.

### CLAD

We found no statistically significant increase in the incidence of CLAD, an accelerated progression to CLAD or a tendency towards a specific CLAD phenotype with EVE. Not unexpectedly, the greater the ventilatory reserve at the time of starting EVE (FEV_1_ and FVC), the less likely the recipient would progress to CLAD.

Traditionally outcomes in LTR with RAS tend to be worse than those with BOS with shortened survival (28). We found on multivariate analysis, patients with BOS unexpectedly had a greater risk of death compared to those with RAS. The potential benefit of EVE in LTR with RAS and the potential mechanisms for these findings warrant further investigation. The development of CLAD has been suggested to be due to chronic fibroblast activation and EVE is known to downregulate fibroblast activity (10). A potential mechanism could be the antifibrotic activity of EVE influencing the chronic fibroblast activation present in CLAD.

Other benefits of EVE may be due to its impact on angiogenesis. Angiogenesis is a complex process in the transplanted organ and involves cellular proliferation, vascular remodelling, and endothelial activation. EVE has anti-angiogenic effects *in vitro* that prevent cellular proliferation, vascular remodelling, and endothelial activation with potential benefits in chronic allograft rejection (9). EVE may indeed have promoted a stabilizing influence on pulmonary function once CLAD was established. These findings also reassure us that there was no long-term clinically significant underlying EVE lung toxicity (29).

On multi-variate analysis, as a time-dependent variable, mycophenolate containing immunosuppressant regimens were associated with improved survival. At our institution, mycophenolate is utilized as a second line agent in sensitized recipients or LTR who are commenced for the management of ACR. Azzola et al. demonstrated that EVE and mycophenolate were the two most potent antifibroproliferative drugs at concentrations achieved clinically (30). Low doses of EVE and mycophenolate can achieve at least 50% inhibition of fibroblast proliferation at therapeutic doses (30). The combination of mycophenolate and EVE may provide a synergistic benefit in LTR with CLAD due to their antifibrotic activity warranting further investigation.

### Preservation of Renal Function

The most common indication for starting on EVE in our cohort was renal preservation due to CNI-related nephrotoxicity. Preservation of renal function is paramount post-LTx as nephrotoxicity is associated with significant morbidity and mortality (31). EVE provides an alternate immunosuppressive agent in the LTx setting with potentially less nephron loss over time from reduced long-term exposure to CNIs (3, 4). Other studies in LTx have investigated the change in renal function parameters following the introduction of EVE (3, 4). Similarly, to these studies we found no increase in renal related mortality with the introduction of EVE as part of maintenance immunosuppressive regimens.

Although on univariate analysis, LTR who started EVE for renal preservation appeared to progress to CLAD faster than those that did not, this was not borne out in multivariate or survival analyses. A possible explanation for the faster time to CLAD is the contribution of renal impairment to lung function decline. It is known that LTR with chronic kidney disease demonstrate abnormalities in lung function including obstructive and restrictive ventilatory defects, and impaired diffusing capacity (32).

Additionally, it is likely that those started on EVE-based regimens due to renal impairment had a period of subtherapeutic CNI levels with the aim of stabilizing renal function in the short-term. These potential prolonged periods of low CNI levels may have led to the immunological risk of periods of suboptimal immunosuppression contributing to an increased risk of CLAD. We would suggest that in those with CNI intolerance, it may be prudent to consider starting EVE earlier rather than continue with subtherapeutic levels of CNI and the risk of inadequate immunosuppression.

### Limitations

Our study has several limitations. Firstly, the findings are retrospective, reflect a single center experience and the EVE prescribing patterns of this center have evolved since 2005. Immunosuppression regimens and target levels would be adjusted over time according to unit protocol and modified further according to response and tolerance. In addition, the study cohort was heterogenous, with indications for EVE use including nephrotoxicity and neurotoxicity and regimen strategies that varied from CNI minimization to elimination.

Secondly, as our center does not routinely monitor TLC this could not be utilized in the definition of RAS and therefore spirometric measures as previously detailed were incorporated into our criteria for RAS diagnosis (21, 23).

### Conclusion

EVE-based maintenance immunosuppression can be successfully and safely be utilized when CNI minimization or elimination is required. Most importantly, our analyses demonstrated that starting EVE does not increase the risk of death or accelerate the progression to CLAD.

## Data Availability

The raw data supporting the conclusion of this article will be made available by the authors, without undue reservation.

## Abbreviations

CMV, cytomegalovirus; eGFR, estimated glomerular filtrate rate; EVE, everolimus; LTR, lung transplant recipient; LTx, lung transplantation; RAS, restrictive allograft syndrome; SOT, solid organ transplant.
